# Social determinants and reflections for the Nursing practice in times of COVID-19*

**DOI:** 10.1590/1518-8345.4907.3443

**Published:** 2021-07-19

**Authors:** Débora de Souza Santos, Nathália de Souza Monezi, Isabeli Karine Martins Castelaneli, Maria Filomena de Gouveia Vilela

**Affiliations:** 1 Universidade Estadual de Campinas, Faculdade de Enfermagem, Campinas, SP, Brazil.

We read with interest the article entitled Effect of income on the accumulated incidence of COVID-19: an ecological study^1^, which indicates, as to April 2020, higher incidence rates in neighborhoods with a high *per capita* income, pointing as hypotheses the greater social isolation in the wealthiest neighborhoods at the beginning of the pandemic and the underreporting in the poor regions.

We share the authors’ concerns^1^ about the severity of the social vulnerability of minority groups, especially the black-skinned population, which has historically been the most affected by respiratory transmission diseases and has less access to health services. More recent studies, conducted from the June and July data, confirm the racial inequalities in the COVID-19 outcomes in the Brazilian pandemic: it was identified that brown skin color/race is the second risk factor for death^2^ and that lethality for pregnant black-skinned women is almost two-fold when compared to white-skinned women^3^. The ethnic disparities were also reported in cities from the United States of America^4^ with a concentration of higher incidence and mortality due to COVID-19 in the Afro-American population.

The concept of vulnerability in health^5^ is complex and encompasses the individual, social and political dimensions, so that the chance of illness is the result of a set of contextual factors, which are related to non-existent or reduced access to fundamental rights. The Social Determinants of Health (SDHs)^5^ express the degrees of vulnerability of the different groups, based on the social and economic conditions, with detriment to the poorest and most peripheral populations. In addition to this situation, there is racism as a structural SDH that accentuates the exclusion of the black-skinned Brazilian population from access to rights^3^.

In a pioneering study led by nurses in Brazil, the disparity observed in the COVID-19 outcomes between white- and black-skinned pregnant and puerperal women was not associated with biological factors^3^. In the absence of clinical differences of greater risk for COVID-19, structural racism as a SDH was pointed out as an explanation for the late arrival of black-skinned women in the services, in worse health conditions, resulting in outcomes twice more tragic for these women^3^.

For Nursing, analyzing the health-disease process with the new coronavirus through the lens of the SDHs^3^, in a country with serious social and ethnic disparities, implies the development of coping policies and practices with a focus on health vulnerability. The challenge is even greater when adding the high rate of underreporting^2^, where the vulnerable groups are the most overlooked^1^.

It should be noted that 2020 was considered the International Year of Nursing, with the launch of the *Nursing Now* global campaign by the World Health Organization. In the context of a pandemic, in which disparities are accentuated, we emphasize the fundamental role of Nursing in the formulation of public policies committed to the reduction of health inequalities, considering its capacity for collaborative and inter-professional work, inter-sectoral action and the use of permanent education for the transformation of work, in the context of intense changes that require sensitivity and creative and relational capacity, attributes historically developed by Nursing.
